# The Application of Mobile fNIRS in Marketing Research—Detecting the “*First-Choice-Brand”* Effect

**DOI:** 10.3389/fnhum.2018.00433

**Published:** 2018-11-01

**Authors:** Caspar Krampe, Nadine Ruth Gier, Peter Kenning

**Affiliations:** Faculty of Business Administration and Economics, Heinrich-Heine-Universität, Düsseldorf, Germany

**Keywords:** fNIRS, first-choice-brand effect, “neuro-marketing”, consumer neuroscience, shopper neuroscience, neuroimaging

## Abstract

Recent research in the field of “neuro-marketing” shows promise to substantially increase knowledge on marketing issues for example price-perception, advertising efficiency, branding and shopper behaviour. Recently, an innovative and mobile applicable neuroimaging method has been proposed, namely functional near-infrared spectroscopy (fNIRS). However, this method is, in the research field of marketing, still in its infancy and is, consequently, lacking substantial validity. Against this background, this research work applied a convergent validity approach to challenge the validity of (mobile) fNIRS in the field of “neuro-marketing” and consumer neuroscience. More precisely, we aim to replicate a robust and well-investigated neural effect previously detected with fMRI—namely the “*first-choice-brand*” effect—by using mobile fNIRS. The research findings show that mobile fNIRS appears to be an appropriate neuroimaging method for research in the field of “neuro-marketing” and consumer neuroscience. Additionally, this research work presents guidelines, enabling marketing scholars to utilise mobile fNIRS in their research work.

## Introduction

During recent decades, substantial milestones have been passed by marketing scholars moving marketing research forward (Eisend, 2015). Although this accumulation of knowledge has increased scholars and practitioners understanding, some marketing issues remain unsolved and might not be explorable using existing marketing methods (Zaltman, 2000; Eisend, 2015). To account for the diminishing utility of existing marketing methods (Eisend, 2015), scholars integrated innovative methods from cognate disciplines. Notably, the discipline of consumer neuroscience, in a business context also known as “neuro-marketing” (Hubert and Kenning, 2008; Harris et al., 2018), promises to substantially increase knowledge of marketing issues, for example price-perception, advertising efficiency, branding, purchase and shopper behaviour (e.g., Kosslyn, 1999; Kenning and Plassmann, 2005; Knutson et al., 2007; Plassmann et al., 2015; Falk et al., 2016; Kühn et al., 2016; Barnett and Cerf, 2017). This progression is predominantly driven by the belief that the utilisation of neuroscientific methods will add supplementary information to existing concepts and theories (Zaltman, 2000; Kenning and Plassmann, 2005; Plassmann et al., 2015). Fortunately, marketing research can greatly benefit from methodological progress in the research field of neuroscience. Mainly because, just recently, a novel neuroimaging method, namely mobile, functional near infrared-spectroscopy (fNIRS), emerged (Kopton and Kenning, 2014).

fNIRS is a relatively new, non-invasive neuroimaging technique that utilises near-infrared light sources able to penetrate human tissue (Ferrari and Quaresima, 2012). More precisely, (mobile) fNIRS uses specific wavelengths of light (760 and 850 nm) to provide a measurement of cerebral oxygenated (oxy-Hb) and deoxygenated haemoglobin, which are the main absorbers of near-infrared light (Kopton and Kenning, 2014), allowing the indirect quantification of neural activity to be measured. There are several fNIRS technologies applied (for further information please see: Scholkmann et al., 2014; Torricelli et al., 2014; Brugnera et al., 2018). In this research work, we used one of the most commonly utilised fNIRS technology, namely the continuous wave (CW) method, which allows to compute changes in oxygenated, deoxygenated and total haemoglobin concentrations from a calculated baseline (Torricelli et al., 2014). There is profound evidence that the fNIRS signal correlates significantly with the functional magnetic imaging (BOLD) signal (Fishburn et al., 2014; Masataka et al., 2015). The spatial resolution and penetration depth of mobile fNIRS is dependent upon the distances between light sources and detectors but generally capable of imaging depths of up to 2 cm (McCormick et al., 1992). This allows the measurement of neural activity in brain regions such as the prefrontal cortex (PFC), which plays a crucial role in consumers cognitive processing such as for example buying decisions (Deppe et al., 2005, 2007; Gonzalez et al., 2005; Knutson et al., 2007; Schaefer and Rotte, 2007; Plassmann et al., 2008; Quaresima and Ferrari, 2016; Goodman et al., 2017).

However, although previous research indicated the validity of fNIRS as a neuroimaging method in various scientific disciplines (Fishburn et al., 2014; Naseer and Hong, 2015; Kim et al., 2016; Werchan et al., 2016), to date, there is very little evidence supporting its utilisation in (neuro-)marketing research.

This is surprising given the fact that especially the application of the *mobile applicable* version of fNIRS might have the potential to overcome or at least reduce one of the major concerns of most neuroimaging techniques—it’s immobility (Arnsten and Goldman-Rakic, 1998; Miyai et al., 2001; Atsumori et al., 2010; Funane et al., 2011; Szalma and Hancock, 2011; Yoshino et al., 2013; Boksem and Smidts, 2015).

However, because mobile fNIRS is still in its infancy, at least in the research field of marketing, this appealing method is lacking substantial validation. To address this issue, we applied a convergent validity approach to challenge the validity of mobile fNIRS. In particular, we strive to replicate a robust and well-investigated neural effect, previously detected with fMRI—namely the “*first-choice-brand*” effect—by using mobile fNIRS.

## Proof of Concept—The Mobile fNIRS Validation Approach

The fact that the validity of mobile fNIRS has, to the present day, never been conventionally challenged in marketing research might be one reason for its limited utilisation. It is, however, fundamental that every novel method provides evidence of its validity specific to the scope of application. When discussing the concept of validity, it is essential to understand that* validity*, in a general sense, determines whether the research method truly measures that which it was intended to measure (Golafshani, 2003). However, in the literature there is a distinction between different kinds or “concepts” of validity that are (Gravetter and Forzano, 2003):

(i) the *predictive validity* which is demonstrated when a measurement accurately predicts behaviour according to a theory;
(ii) the *construct validity* which requires that the measurements obtained from a measurement procedure behave exactly the same as the variable itself;
(iii) the *divergent validity* which is demonstrated by using two different methods to measure two different constructs, accordingly there should be no or only a little relationship between the measurement obtained from the two different constructs when they are measured by the same method; and
(iv) the *convergent validity* which is demonstrated by a strong relationship between the scores obtained from two different methods of measuring the same construct (Gravetter and Forzano, 2003).

That said, it should be evident that scholars can choose from a set of validation approaches. For example, to validate mobile fNIRS scholars can follow the predictive validity approach (i), hypothesising that mobile fNIRS is able to quantify a particular neural brain activity based on a stimulus presented. Scholars could therefore assume, based on literature and theory, that visual stimuli lead to neural brain activity in the visual cortex, by testing this hypothesis utilising mobile fNIRS, scholars will be able to make an assumption about the validity of its utilisation.

By utilising the construct validity approach (ii), scholars can validate a neuroimaging method such as mobile fNIRS by showing that the same method can differentiate between two different and well investigated scientific constructs. Scholars could therefore, based on the knowledge that vision and motoric processes are located in different brain regions, indicate that it is possible, by means of mobile fNIRS, to distinguish neural cortical activity of visual and motoric stimuli, investigating the same construct namely neural activity. Consequently, depending upon the stimulus only one related brain region should be activated, allowing a proposition to be made about the operating principles of mobile fNIRS.

Moreover, scholars might choose to explore the validity of a neuroimaging method by utilising a divergent validity approach (iii), comparing two different deviating methods investigating two different entities. As a result, both methods should have contradictory outcomes.

Finally, in order to validate mobile fNIRS in a specific scope of application (as for example in the field of marketing), scholars could choose for a convergent validity approach (vi), replicating a robust and previously explored (neural) effect whilst employing an existing and already validated neuroimaging technique. By applying the same research paradigm as used in a previous research work, scholars could compare data acquired with an innovative and a validated (neuroimaging) method, verifying the innovative methods in the scope of application whilst exploring an existing, validated entity.

Taking the aforementioned concepts into account, we chose the convergent validity approach in order to explore whether mobile fNIRS is a suitable neuroimaging method also for marketing research and consumer neuroscience. The reason for this relies on the unique characteristics of the marketing relevant, neural effect of the “*first-choice-brand*” (Deppe et al., 2005). Based on its two interrelated sub-effects, the “*first-choice-brand*” effect is capable of providing information about potentialities as well as limitations directly related to mobile fNIRS and its technical capabilities, e.g., when it comes to measure subjacent brain regions. Insights that are solely explorable whilst utilising a convergent validity approach.

Consequently, based on scientific evidence and based on the technical capabilities of mobile fNIRS, we aim to *partly* replicate the “*first-choice-brand*” effect (Deppe et al., 2005; Koenigs and Tranel, 2007).

This specific brand-related effect, was first reported by Deppe et al. (2005), indicating that participants have distinctive neural activity in brain regions of the PFC whilst making a binary buying decision when their favoured brand (*first-choice-brand, FCB*) is involved. The “*first-choice-brand*” effect, which will be discussed in the next section, was found in several subsequent studies and seems to be a robust neural effect.

## Theoretical Background—the “*First-Choice-Brand*” Effect

In essence, the “*first-choice-brand*” effect consists of two interrelated sub-effects. The *first sub-effect* is characterised by an increased neural activity in the ventromedial PFC (vmPFC), a subjacent medial brain region involved in processing of emotions, episodic memory retrieval and self-reflection during decision making (Deppe et al., 2005), displaying self-referential processes during first choice brand decision-making. The *second sub-effect* is characterised by reduced neural activity in the dorsolateral PFC (dlPFC), a brain region generally associated with working memory, inductive reasoning, planning, cognitive control, strategy-based reasoning, judgments and reasoning-based decision making (Braver et al., 1997; Courtney et al., 1997; Pochon et al., 2001; Kroger et al., 2002; Manes et al., 2002; Raye et al., 2002; Curtis and D’Esposito, 2003; Deppe et al., 2005). The reduced neural activity of the dlPFC might, potentially, indicate that strategy-based reasoning and judgments are reduced when participants are exposed to their favoured “*target*” brand compared to non-preference decision making trials (Deppe et al., 2005; Koenigs and Tranel, 2007; Schaefer and Rotte, 2007), allowing the consumers to take a quicker, straightforward and less complex decision when their individual target brand is present—a neural effect which is also called cortical relief effect (Kenning et al., 2002).

Based on the technical parameters of mobile fNIRS, in particular its spatial resolution and penetration depth (McCormick et al., 1992), mobile fNIRS is not capable of measuring subjacent medial brain regions lying deep in the brain. Consequently, we do expect that mobile fNIRS is solely able to partly replicate the “*first-choice-brand*” effect, namely only the *second sub-effect*. Against this background, we investigate the following hypotheses:

- *H_1_*: mobile fNIRS is capable of measuring decreased neural activity in the dlPFC when consumers’ decision making is associated with their first-choice-brand.
- *H_2_*: mobile fNIRS is not capable of measuring increased neural activity in the vmPFC when consumers’ decision making is associated with their first-choice-brand.

## Method

### Participants

In order to empirically test our hypotheses, a total number of *N* = 42 (Friston, 2003) right-handed (e.g., Toga and Thompson, 2003), female household running participants was recruited in order to take part in a fNIRS-experiment at University of Düsseldorf, Germany (*M* = 38.07, *SD* = 11.13 years of age, *M* = 1,725.49, *SD* = 879.28 net income in Euro). With regard to the fact that women are more frequent customers of grocery retailers and are, therefore, more frequently exposed to brand related decisions, only household running, female participants were recruited for this experiment (Rampl et al., 2012). All participants had normal vision and no history of neurological disorder and were informed about the nature of the experiment as well as the operating mode of mobile fNIRS, before the written informed consent was signed^1^. All subjects gave written informed consent in accordance with the Declaration of Helsinki. Subsequently, to increase their involvement, participants were requested to imagine that acquaintances had asked them to buy a high-quality filter coffee for their cream-tea appointment at the weekend. As they wanted to make a good impression, the participants needed to choose, whilst making their buying decision, the coffee brand which had, in their opinion, the highest quality. After completing the experiment, participants were asked to sort all coffee brands according to their preferences and buying intention. Based on the individual ranking, the selection of the target brand group (TBG) and the diverse brand group (non-TBG) was performed (Deppe et al., 2005). Only participants who rated the target coffee brand (the market leader) as their favourite brand, were assigned to the TB-group. This is essential in order to analyse the data, since the reduced dlPFC activity is only hypothesised for the TBG in comparison to the non-TBG. Furthermore, in post-experiment-interviews participants were enquired about their (filter coffee) shopping behaviour, asking if they are aware of the target brand as used in the experiment. This is inevitable to experience and consequently to display the “*first-choice-brand*” effect in the designed experiment, utilising mobile fNIRS. Only when participants indicated that they have never bought filter coffee before, they were excluded from further analysis. Following this procedure 10 participants were excluded, resulting in a sample size of *N* = 32 (*M* = 37.97, *SD* = 10.97 years of age) of which 16 participants selected the predefined target brand (T) as their favoured brand.

### Task Procedure

**Figure 1:**
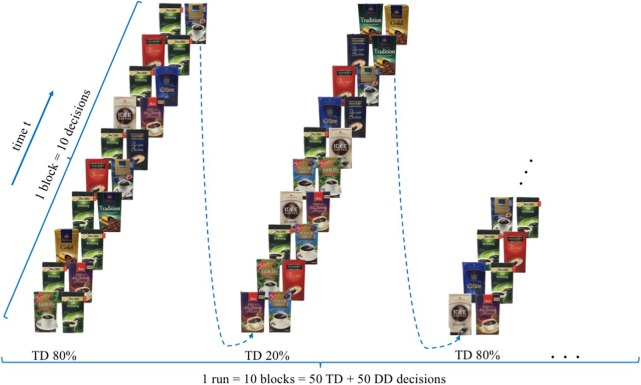
Experimental design—modified version of the paradigm created by Deppe et al. (2005).

The experimental task aims to examine if the presence of a filter coffee target brand, evoke a reduced neural, bilateral activity in brain regions ascribed to the dlPFC for participants who rated the target brand, *ex post*, as their favoured brand, measurable with the use of mobile fNIRS. Extending previous research (Deppe et al., 2005; Koenigs and Tranel, 2007; Schaefer and Rotte, 2007), participants were shown 100 different buying decisions scenarios in the presence or absence of a specific target brand (T) in which participants had to take a binary buying decision. “Jacobs Krönung” as German market leader was defined, *a priori*, as the target brand (T). The other brands were classified as diverse (D), resulting in a binary decision-making set of either TD (TD decision = target brand vs. diverse brands) or DD (DD decision = diverse brand vs. diverse brands) decisions. All trials were presented in a 10 × 10 event-related design, whereby in alternating order two types of trial composition were displayed. The compositions consisted either of 8 DD and 2 TD (20% TD) or 2 DD and 8 TD (80% TD) decisions, whereby the order of DD and TD decisions was randomised (please see Figure 1). For each of the 100 trials, participants had the option of two different coffee brands, which were presented on a computer screen, lasting for 3 s. The trials were separated from each other by means of jittered fixation cross lasting 4 to 6 s. For each single decision trial, participants were requested to decide mentally, were no manual response was required, which of the two displayed coffee brands they would like to buy.

No resting condition was implemented between the blocks, since participants needed merely 800 s (13.3 min) to complete the whole paradigm.

To assure the same environmental circumstances for every participant, the temperature and light conditions were kept equally and the background noises were kept to a minimum. Throughout the experimental task the experimenter left the room, but re-entered the room after participants indicated that they have completed the experimental task.

## Data Collection, (Pre-)Processing and Results

### Data Collection

**Figure 2:**
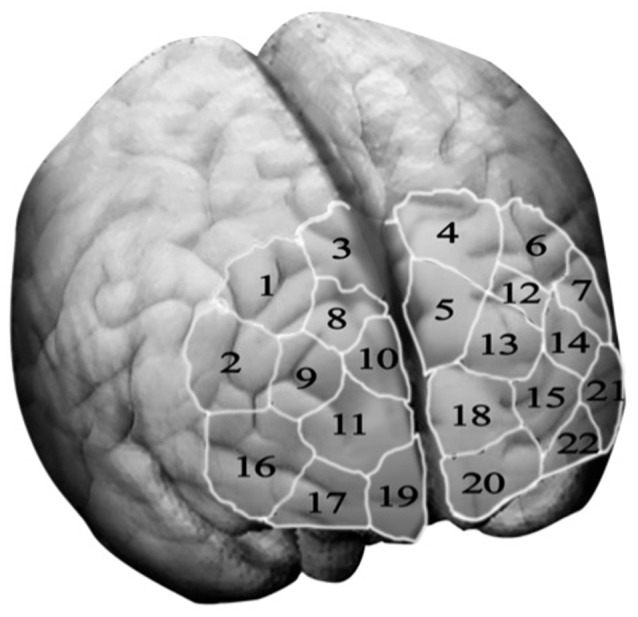
Topographical layout—The topographical layout is a schematic representation of the measurements sites. It integrates 22 channel, each corresponding to a particular brain region of the three sub-regions of the prefrontal cortex, namely the dorsolateral prefrontal cortex (key channels 9 and 14), the orbitofrontal cortex (key channels 19 and 20) and the motor cortex (key channels 1, 3, 4 and 6).

Optical signals were recorded on a two-wavelength (760 and 850 nm) continuous-wave fNIRSport-System (NIRx Medical Technologies, Berlin, Germany^2^). Data was collected from detectors in parallel at a sampling rate of 7.81 Hz. The optical channels were comprised of eight sources and eight detectors. Optodes and diodes are separated from each other by a distance of 3 cm in order to guarantee signal quality. Participants are fitted with a headband, covering most of the PFC in particular bilateral orbitofrontal cortex (OFC), bilateral dlPFC and bilateral premotor cortex. To ensure that the headband is located according to the anatomical brain structures of the participants, the craniometric point of the nasion, where the top of the nose meets the ridge of the forehead, was used to assure comparability between participants. Based on this configuration the “topographical layout,” a schematic representation of the measurements sites, integrating 22 channels was designed, allowing to measure cortical neural activity of the PFC and its sub-regions as previously described (please see Figure 2). The NIRS-Star software package (version 14.2) was used to check for signal quality and data collection.

### Data (Pre-)Processing

Before further analysis, the collected fNIRS raw data were pre-processed. Therefore, to smooth the raw data a band-pass filtered (high/low frequency filter) was applied in order to control for artefacts that might interfere with the measurement of the intended effects, as for examples the heartbeat or strong and abrupt head movements. The lower cut off frequency value was set to 0.01 Hz, whereas the higher cut off frequency value was set to 0.2 Hz.

Raw optical density signals were converted to haemoglobin concentration changes using the modified Beer-Lambert law (Delpy et al., 1988; Kocsis et al., 2006; Kopton and Kenning, 2014; Scholkmann et al., 2014) within the NIRx Software package (NIRx Medical Technologies, Berlin, Germany^2^). The parameters used to compute the haemodynamic states were set as follows, the distance of the first channel was set to 3 cm, the wavelengths were specified to values of 760 and 850 nanometre and the associated pathlength factor (DPT) was set to 7.25 for the wavelength of 760 nm and 6.38 for the wavelength of 850 nm, in accordance with commonly utilised values reported in literature (Essenpreis et al., 1993; Kohl et al., 1998; Zhao et al., 2002). As the oxy-Hb signal has been shown to correlate with cerebral blood flow better than the deoxygenated signal (Hoshi et al., 2001), the analysis concentrates on the oxy-Hb signal.

**Figure 3:**
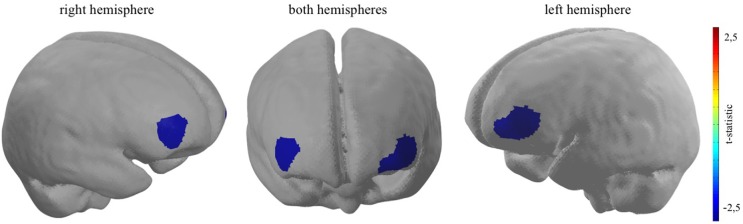
Bilateral dlPFC decreased neural activity when participants who rated the target brand first are confronted with a TD decision. *Channel 16*: *t*_(31)_ = −2.42, *p* < 0.05; *channel 21*: *t*_(31)_ = −2.74, *p* < 0.01; *channel 22*: *t*_(31)_ = −2.49, *p* < 0.05.

For every participant, a general linear model (GLM) was set up to model neural activity during the experimental task. The picture period, displaying the different coffee brands, was modelled separately for TD-trials and DD-trials, adding up to two event-related regressors together with an additional error term at the end (*Y*_j_ = *x*_j1_*β*_1_ + *x*_j2_*β*_2_ + ε_j_). Each time course was further corrected for serial correlations such as physiological noise sources, modulating the stimulus onsets convolved by a haemodynamic response function (Worsley and Friston, 1995). No contrast was calculated for every participant individually (on within-subject-level). However, in order to investigate the estimated effects on group-level (between-subject-level), two groups were, based on the *ex post* conducted ranking of the coffee brands, created. Following the original article by Deppe et al. (2005) only participants who rated the target brand as their favourite brand, were assigned to the TB-group. T-contrasts were used to generate statistical parametric maps of activation by contrasting TD decisions of the TBG (*N* = 16) in comparison to TD decisions of the diverse brand group (non-TBG, *N* = 16). A t-contrast activation map of the neural PFC activity was plotted on a standardised brain. The activation map threshold was set to a *p*-value of *p* < 0.05 (please see Figure 3).

### Data Results

As hypothesised, the results show significant bilateral cortical dlPFC decreased neural activity when participants take TD decisions, contrasting the TB-group and the non-TB group on a significant level of *p* < 0.05. Giving evidence for the second sub-effect, the cortical relief effect (Schaefer and Rotte, 2007; Kenning et al., 2002) in the TB-group, which was solely determined by the presence of the participant’s most favoured target brand during binary buying decisions. More precisely, channel 16 (*t*_(31)_ = −2.42, *p* < 0.05, *d* = −0.86), channel 21 (*t*_(31)_ = −2.74, *p* < 0.01, *d* = −0.97) and channel 22 (*t*_(31)_ = −2.49, *p* < 0.05, *d* = −0.88), which are localised in dlPFC brain regions indicate a reduced neural activity when participants, who rated the target brand as their favoured brand, had to decide between a target and diverse brand in a binary buying decision task (please see Figure 3). Furthermore, as the effect size, measured with Cohen’s *d*, exceed the value of 0.8 for all three reported effects, the magnitude of the measured effects can be defined as strong (Cohen, 1988). Moreover, confirming H_2_ no significant increase in neural activity could be pictured in brain regions ascribed to the vmPFC, when the target brand was present, given participants ranked the target brand first (for detailed information, see Supplementary Appendix 1.1–1.4).

## Mobile fNIRS—A Validated Neuroimaging Method?

The results of our study clearly support the assumption that mobile fNIRS has, in principle, the ability to assist scholars and marketers to enlarge knowledge, methods and analyses from extant approaches of consumer research, developing marketing theory and consumer research findings.

Whilst our research results indicated, in line with previous work (Deppe et al., 2005), that a participants’ first-choice brand decreases the neural activity in brain regions ascribed to the dlPFC, simplifying the participants buying decision (Schaefer and Rotte, 2007), our research results also indicate some limitations of mobile fNIRS when it comes to measure subjacent brain region such as (parts of) the vmPFC (Koenigs and Tranel, 2007). Based on our results and previous research findings (McCormick et al., 1992) it should therefore be evident that mobile fNIRS is not always a suitable neuroimaging method or even a panacea; and cannot be applied when the research focus relies on, for example, subjacent brain regions. An example in this regard might be the measurement of emotional and perception processes such as price (fairness) perception (Knutson et al., 2007; Linzmajer et al., 2014). Given the fact that emotional and perception processes find its neural origin in “deeper” brain regions such as e.g., the hippocampus, the insula, the nucleus accumbens and/or the amygdala, mobile fNIRS with its technical capabilities, might currently not be able to shed light on these processes. Moreover, next to its spatial resolution, the temporal resolution of mobile fNIRS seems to be lower in comparison to for example electroencephalography (EEG), but seems to outperform the temporal resolution of fMRI (Wilcox and Biondi, 2015).

By keeping in mind that every neuroimaging method has its advantages and disadvantages, it should be noted that mobile fNIRS might have, in particular, the ability to: (1) minimise purchase and running costs; (2) increase the ecological validity, due to its potential mobile usage; and (3) exploit an extended sample, integrating participants that have been excluded earlier because of physical criteria (see Kopton and Kenning, 2014). Against this background, scholars and marketers should carefully select and verify the methodological instrument they would like to apply to answer a specific research focus. In the next section, we, therefore, aim to provide marketing scholars a short guideline of how to apply mobile fNIRS to their own research. In particular, we will try to answer the following questions: *If fNIRS is the answer, what should be the question? When should mobile fNIRS be applied in marketing research? And, finally, how should mobile fNIRS be applied (in a real world situation) and how to analyse the generated data?*.

## Mobile fNIRS—A Short Guideline for Marketing Research

### If fNIRS Is the Answer, What Should be the Question?

As mentioned before, it should be evident that neuroscientific methods do not guarantee intended results and, consequently, increase the explained variance of a scientific entity. Instead, sometimes it is needless, costly and risky to utilise neuroscientific methods to answer a marketing related research question. Consequently, scholars need to be aware, *ex ante*, whether a neuroscientific tool has the potential to increase their understanding of a marketing related construct and can, therefore, add to existing marketing theories.

In order to illuminate whether mobile fNIRS can add to marketing research, scholars should ask themselves two additional questions, keeping the capabilities of a mobile fNIRS in mind: (i) How is information processing implemented within the brain and how is this related to a particular entity/ability? and (ii) when are particular processes and brain structures invoked? (Kosslyn, 1999).

These essential questions might be answered by conducting a comprehensive literature review in which scholars assimilate crucial information about their research focus and its connection to cognitive processes manifested in particular brain structures, combining both, the marketing and neuroscientific knowledge in one nomological network.

For example, if a marketing scholar is interested in emotional processes associated with a certain product characteristic (e.g., a car design), it should be evident that mobile fNIRS is, at the present time, not suitable to measure emotional processes, which find their neural origins in subcortical brain regions like the amygdala or the hippocampus (McCormick et al., 1992). Moreover, it is essential for marketing scholars to assure themselves that a given research question cannot be answered with existing marketing research methods, which might be more cost-effective (Ariely and Berns, 2010) and/or easier to administer (Dimoka et al., 2012).

As mobile fNIRS opens up the ability to gather neural data in a naturalistic environment, increasing the ecological validity, it should also be evident that the temporal resolution of mobile fNIRS might sometimes, depending on the research approach, be a limitation. This might especially be the case when a stimulus occurrence is uncontrolled in a naturalistic environment.

Against this background, marketing scholars should refrain from utilising mobile fNIRS when its application does not provide essential information above and beyond information quantifiable with existing measurements. Furthermore, they should refrain when no further information about the underlying cognitive processing mechanism, no information in regard to particular processes, the related brain structures and/or information about the temporal function of the process is provided.

### When Should Mobile fNIRS be Applied in Marketing Research?

After the question “whether” mobile fNIRS generated knowledge can increase the explained variance of a marketing relevant entity is answered, scholars should ask themselves “when” to apply mobile fNIRS. Due to the rapid proliferation of neuroscientific methods and techniques, the absence of clear guidance how to conduct high-quality, user-oriented consumer neuroscience research and a possible ignorance of the added value of the integration of neuroimaging methods, it could become difficult for marketing scholars to decide, based on their research focus, which neuroscientific method to employ. So, scholars need to be aware about the technical capabilities of a given neuroscientific method. In line with this, neuroscientific methods could be categorised according to three dimensions, the temporal resolution, the spatial resolution and whether a neuroscientific method is portable or not.

**Figure 4:**
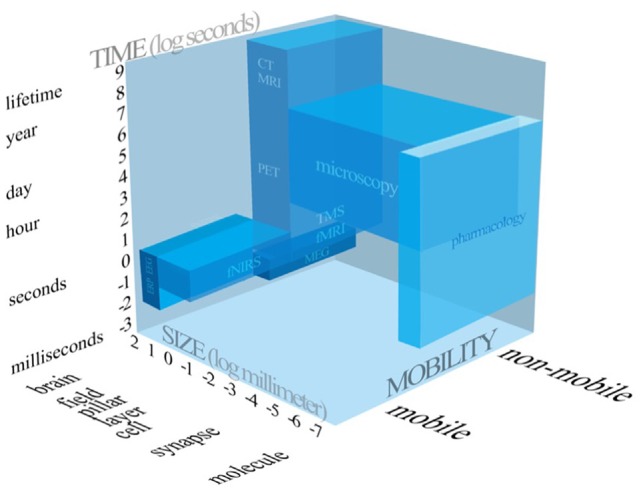
Systematic representation of the neuroscientific tools applied in consumer neuroscience and related research fields.

Figure 4 compares the frequently applied neuroscientific methods, intends to summarise all three dimensions scholars need to be aware of, when applying neuroscientific methods.

Accordingly, scholars have to choose wisely, based on their research focus, whether to apply mobile fNIRS or not. As mentioned before, based on our research results it should be evident that mobile fNIRS is not able to measure the whole brain, but is capable of imaging depths of up to 2 cm (McCormick et al., 1992) of the human cortex. Consequently, subjacent medial brain regions cannot be measured by utilising mobile fNIRS. Hence, if the research focus rely on cognitive processes which are related to subjacent brain region, scholars might apply another neuroimaging methods, such as fMRI.

**Figure 5:**
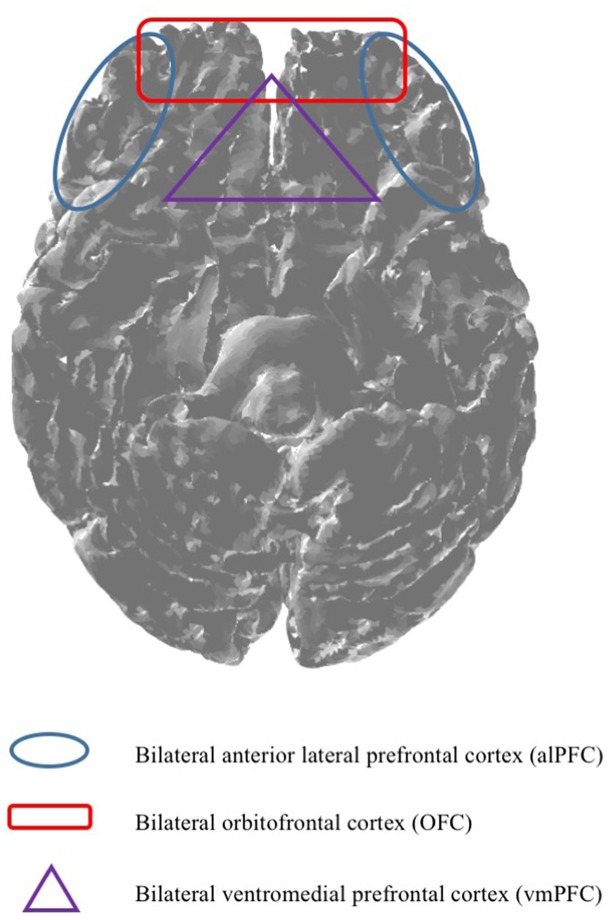
Based on the technical capabilities, mobile fNIRS is capable of imaging depths of up to 2 cm (McCormick et al., 1992). Against this background, it is essential to predefine brain regions, *ex ante*, indicating which brain regions are measurable by utilising mobile fNIRS.

Even though, previous research indicated that fNIRS is capable of measuring brain regions such as for example the left anterior OFC (Ernst et al., 2013). There seems to be uncertainty regarding the technical capabilities of mobile fNIRS. Against this background, based on the fact that the previously mentioned brain regions are only vaguely defined and often incorporate wide areas of the PFC, it is particularly difficult for scholars to specify if mobile fNIRS is also capable of measuring subjacent brain regions which may interest them, such as the vmPFC. Consequently, based on the insufficient classification of cortical brain regions, our research work provides, based on our research results, a classification map (please see Figure 5), giving scholars the opportunity to decide if mobile fNIRS is able to measure a pre-defined region of the brain in which they are interested, or not. More precisely, a brain region such as the vmPFC might not be explorable (purple triangle) based on the technical capabilities of mobile fNIRS. Whereas brain regions which are located near the surface of the cortex, such as the OFC, alPFC and dlPFC, might be explorable with mobile fNIRS (red quadrant and blue circle).

### How Should Mobile fNIRS be Applied (in a Real World Situation) and How Should the Generated Data be Analysed?

Mobile fNIRS has, in comparison to other neuroimaging methods, the advantage that it is portable administrable, allowing scholars to utilise mobile fNIRS in real world scenarios. Therefore, in order to collect real world neural data to answer a particular research question, marketing scholars should follow a three-step approach.

The first step is the preparation of the experimental setting—the environment. This is indispensable based on the temporal resolution of mobile fNIRS, which requires the appearance of a stimuli for around 2 s to 3 s in order to measure the associated neural response. Given that the ultimate goal is to measure consumers in a real-world situation, such as at the point-of-sale (PoS), at the moment it is still necessary to prepare the environmental settings in order to account and control for potential confounding effects and to guarantee the perception of a stimuli that might be manipulated in a research paradigm.

The second step is the acquisition of data. In order to collect mobile fNIRS data to answer a particular research question, participants need to be equipped with a headband or a cap, comprising light sources and detectors that cover parts or the whole cortex. After the headband or cap is placed on the cortex scholars have to calibrate it to make sure that the signal quality is to their satisfaction. To check for the fNIRS signal quality, scholars could use several, mostly with the hardware delivered software packages^3^. Before starting the calibration to check for the fNIRS-signal quality, scholars should eliminate external light sources which might interfere with the fNIRS-signal by protecting the measured brain region with a light impermeable cap. Moreover, before starting the experiment participants should be informed that strong and abrupt (head)movements during data collection should be avoided. This is essential in order to guarantee appropriate fNIRS data quality and prevent strong (movement) artefacts.

In comparison to a stationary conducted fNIRS experiment, the implementation of mobile fNIRS might be even more challenging, as it implies an adequate preparation of the experimental setting in which the data acquisition takes place. Moreover, given that it is rather difficult to define the occurrence of stimuli in a mobile, naturalistic experiment beforehand, it is necessary to combine mobile fNIRS with other neurophysiological methods such as eye-tracking to control for external, environmental cues. This is also relevant for the data analysis of fNIRS experiments conducted in the field, as it takes significantly more time and effort to analyse the data as the stimuli onsets have to be defined *ex post* and with the help of another neurophysiological methods such as e.g., eye-tracking. Nevertheless, based on our research work and recent research findings (Krampe et al., 2018a,b), it should be evident that the advantages of conducting mobile fNIRS experiment may exceed potential disadvantages.

The third step in this process is the data analysis. Before starting the data analysis, scholars need to define task-specific events based on the *ex-ante* established paradigm or manually by defining the onsets (time a stimulus occurred) and length of the stimulus (time a stimulus was present) for every event respectively. This is essential in order to analyse the data statistically. Similar to other neuroimaging data analysis procedures, for example fMRI, the fNIRS analysis process may be subdivided into several components.

First, scholars need to check the signal quality of every diode and optode previously defined in a topographic map of the cortex (Figure 2). Second, irrelevant time series might be truncated in order to exclude time intervals from further consideration, which are not relevant to answer a particular research question. Third, scholars might remove discontinuities and spike artefacts from the data time series, which are clearly and apparently qualified as confounding effects. Thereby, abnormalities that have two or more adjacent channels with *t*-values over three standard deviations from the group average (Fishburn et al., 2014) or which indicate significantly more spike artefacts might be excluded from further analysis. Fourthly, scholars should apply a high, low or band-pass frequency filter in order to smooth the fNIRS data time series. Thereafter, scholars need to decide which light signal (alternatively raw data) they would like to investigate. As the oxy-Hb signal has been shown to correlate with cerebral blood flow better than the deoxygenated signal (Hoshi et al., 2001), most of the fNIRS data analysis focuses on the oxy-Hb light signal. It should, however, be evident that mobile fNIRS is capable of examining raw data, oxygenated, deoxygenated and total haemoglobin concentrations, which is one advantage in comparison to other neuroimaging methods. Thereafter, scholars might convert the raw near-infrared light absorption and attenuation data into oxy-, deoxy- and/or total-haemoglobin concentrations. The most common used algorithm for this progression is the modified Beer-Lambert law (Kocsis et al., 2006; Kopton and Kenning, 2014; Scholkmann et al., 2014), which is integrated in the previously described “NirsLab” toolbox, containing several parameters as described in the data analysis section above.

Once the data have been processed, scholars might analyse the haemodynamic-state time series, as defined before based on the *ex-ante* established paradigm, on within-session and/or within-subject level or across multiple sessions or between-subject level. Thereby, a GLM on individual level will be set up to model neural activity during the experimental task, based on the predefined stationary or mobile paradigm.

Finally, to identify the underlying brain regions involved, statistical results are depicted on a standardised brain to visually locate the neural activation patterns and interpret them. Scholars should be very careful about the localisation and designation of the associated brain regions, encouraging scholars to apply the previously introduced classification map of the PFC.

## Conclusion

Returning to the research questions—is mobile fNIRS a valid neuroimaging method for “neuro-marketing” and consumer neuroscience?—we suggest, the answer is “yes, in principle but.” confirming that mobile fNIRS is in some situations and circumstances an appropriate neuroimaging method able to expand knowledge on several marketing research related issues. Thereby, mobile fNIRS has a good temporal resolution but is restricted in its spatial resolution.

By keeping in mind that there are various mobile fNIRS technologies, which might also be applied in the research field of marketing, recent research demonstrated the usability of smaller, two-channel, portable fNIRS devices and its utility in order to investigate emotional and/or stress processes (Brugnera et al., 2017, 2018; Adorni et al., 2018), indicating the proliferous, ongoing technological progression of mobile fNIRS and its potential for marketing research.

Against this background and based on our research findings, we encourage marketing research to apply mobile fNIRS, following our and already established guidelines (Brouwer et al., 2015; Plassmann et al., 2015), whenever the immobility of another method becomes an issue and when previous research indicates that cortical, near surface brain regions are involved.

## Author Contributions

CK, as first author, conducted the study, performed the data analysis and wrote the manuscript. NG participated in carrying out the study and performed the data analysis. PK helped the first author in developing the study and participated in writing the manuscript. All authors read and approved the final manuscript.

## Conflict of Interest Statement

The authors declare that the research was conducted in the absence of any commercial or financial relationships that could be construed as a potential conflict of interest.

## Footnotes

^1^Mobile near-infrared spectroscopy is a non-invasive method, simply projecting (near-infrared) light through the sculp. It is therefore with no advantages or disadvantages associated. Moreover, the utilised stimuli material integrated only coffee brands to which participants are confronted in every German grocery store. Consequently, an ethical approval for conducting the given experiment was not necessary. However, it should be evident that the experiment was conducted according to existing ethical standards (APA’s Ethics Code and in compliance with the Declaration of Helsinki). Therefore, before the experiment started, participants were informed about the nature of the experiment as well as the operating mode of mobile fNIRS. Subsequently, participants were asked if they would like to voluntarily participate in the experiment and informed that they can stop the experiment at any time without any reasons, consequences and/or disadvantages.

^2^http://nirx.net

^3^https://nirx.net/nirstar-1/

## References

[B1] Adorni R. Brugnera A. Gatti A. Tasca G. A. Sakatani K. Compare A. (2018). Psychophysiological responses to stress related to anxiety in healthy aging. J. Psychophysiol. [Epub ahead of print]. 10.1027/0269-8803/a000221

[B2] Ariely D. Berns G. S. (2010). Neuromarketing: the hope and hype of neuroimaging in business. Nat. Rev. Neurosci. 11, 284–292. 10.1038/nrn2795 20197790 PMC2875927

[B3] Arnsten A. F. Goldman-Rakic P. S. (1998). Noise stress impairs prefrontal cortical cognitive function in monkeys: evidence for a hyperdopaminergic mechanism. Arch. Gen. Psychiatry 55, 362–368. 10.1001/archpsyc.55.4.362 9554432

[B4] Atsumori H. Kiguchi M. Katura T. Funane T. Obata A. Sato H.. (2010). Noninvasive imaging of prefrontal activation during attention-demanding tasks performed while walking using a wearable optical topography system. J. Biomed. Opt. 15:046002. 10.1117/1.3462996 20799804

[B5] Barnett S. B. Cerf M. (2017). A ticket for your thoughts: method for predicting content recall and sales using neural similarity of moviegoers. J. Consum. Res. 44, 160–181. 10.1093/jcr/ucw083

[B6] Boksem M. A. Smidts A. (2015). Brain responses to movie trailers predict individual preferences for movies and their population-wide commercial success. J. Mark. Res. 52, 482–492. 10.1509/jmr.13.0572

[B7] Braver T. S. Cohen J. D. Nystrom L. E. Jonides J. Smith E. E. Noll D. C. (1997). A parametric study of prefrontal cortex involvement in human working memory. Neuroimage 5, 49–62. 10.1006/nimg.1996.0247 9038284

[B8] Brouwer A.-M. Zander T. O. van Erp J. B. Korteling J. E. Bronkhorst A. W. (2015). Using neurophysiological signals that reflect cognitive or affective state: six recommendations to avoid common pitfalls. Front. Neurosci. 9:136. 10.3389/fnins.2015.00136 25983676 PMC4415417

[B9] Brugnera A. Adorni R. Compare A. Zarbo C. Sakatani K. (2018). Cortical and autonomic patterns of emotion experiencing during a recall task. J. Psychophysiol. 32, 53–63. 10.1027/0269-8803/a000183

[B10] Brugnera A. Zarbo C. Adorni R. Tasca G. A. Rabboni M. Bondi E.. (2017). Cortical and cardiovascular responses to acute stressors and their relations with psychological distress. Int. J. Psychophysiol. 114, 38–46. 10.1016/j.ijpsycho.2017.02.002 28174110

[B11] Cohen J. (1988). Statistical Power Analysis for the Behavioral Sciences. 2nd Edn. Hillsdale, NJ: Erlbaum Associates.

[B12] Courtney S. M. Ungerleider L. G. Keil K. Haxby J. V. (1997). Transient and sustained activity in a distributed neural system for human working memory. Nature 386, 608–611. 10.1038/386608a0 9121584

[B13] Curtis C. E. D’Esposito M. (2003). Persistent activity in the prefrontal cortex during working memory. Trends Cogn. Sci. 7, 415–423. 10.1016/s1364-6613(03)00197-9 12963473

[B14] Delpy D. T. Cope M. van der Zee P. Arridge S. Wray S. Wyatt J. (1988). Estimation of optical pathlength through tissue from direct time of flight measurement. Phys. Med. Biol. 33, 1433–1442. 10.1088/0031-9155/33/12/008 3237772

[B15] Deppe M. Schwindt W. Kugel H. Plassmann H. Kenning P. (2005). Nonlinear responses within the medial prefrontal cortex reveal when specific implicit information influences economic decision making. J. Neuroimaging 15, 171–182. 10.1177/1051228405275074 15746230

[B16] Deppe M. Schwindt W. Pieper A. Kugel H. Plassmann H. Kenning P.. (2007). Anterior cingulate reflects susceptibility to framing during attractiveness evaluation. Neuroreport 18, 1119–1123. 10.1097/wnr.0b013e3282202c61 17589310

[B17] Dimoka A. Banker R. D. Benbasat I. Davis F. D. Dennis A. R. Gefen D. (2012). On the use of neurophysiological tools in IS research: developing a research agenda for NeuroIS. MIS Q. 36, 679–702. 10.2139/ssrn.1557826

[B18] Eisend M. (2015). Have we progressed marketing knowledge? A meta-meta-analysis of effect sizes in marketing research. J. Mark. 79, 23–40. 10.1509/jm.14.0288

[B19] Ernst L. H. Plichta M. M. Lutz E. Zesewitz A. K. Tupak S. V. Dresler T.. (2013). Prefrontal activation patterns of automatic and regulated approach-avoidance reactions-a functional near-infrared spectroscopy (fNIRS) study. Cortex 49, 131–142. 10.1016/j.cortex.2011.09.013 22036575

[B20] Essenpreis M. Elwell C. E. Cope M. Van der Zee P. Arridge S. R. Delpy D. T. (1993). Spectral dependence of temporal point spread functions in human tissues. Appl. Opt. 32, 418–425. 10.1364/ao.32.000418 20802707

[B21] Falk E. B. O’Donnell M. B. Tompson S. Gonzalez R. Dal Cin S. Strecher V.. (2016). Functional brain imaging predicts public health campaign success. Soc. Cogn. Affect. Neurosci. 11, 204–214. 10.1093/scan/nsv108 26400858 PMC4733336

[B22] Ferrari M. Quaresima V. (2012). A brief review on the history of human functional near-infrared spectroscopy (fNIRS) development and fields of application. Neuroimage 63, 921–935. 10.1016/j.neuroimage.2012.03.049 22510258

[B23] Fishburn F. A. Norr M. E. Medvedev A. V. Vaidya C. J. (2014). Sensitivity of fNIRS to cognitive state and load. Front. Hum. Neurosci. 8:76. 10.3389/fnhum.2014.00076 24600374 PMC3930096

[B24] Friston K. J. (2003). “Introduction: experimental design and statistical parametric mapping,” in Human Brain Function, 2nd Edn., eds Frackowiak R. S. Friston K. Frith C. D. Dolan R. Price C. Zeki S. (Cambridge, MA: Academic Press), 599–632.

[B25] Funane T. Kiguchi M. Atsumori H. Sato H. Kubota K. Koizumi H. (2011). Synchronous activity of two people’s prefrontal cortices during a cooperative task measured by simultaneous near-infrared spectroscopy. J. Biomed. Opt. 16:077011. 10.1117/1.3602853 21806291

[B26] Golafshani N. (2003). Understanding reliability and validity in qualitative research. Qual. Rep. 8, 597–606. Avaliable online at: http://citeseerx.ist.psu.edu/viewdoc/download?doi=10.1.1.461.9549&rep=rep1&type=pdf

[B27] Gonzalez C. Dana J. Koshino H. Just M. (2005). The framing effect and risky decisions: examining cognitive functions with fMRI. J. Econ. Psychol. 26, 1–20. 10.1016/j.joep.2004.08.004

[B28] Goodman A. M. Wang Y. Kwon W.-S. Byun S.-E. Katz J. S. Deshpande G. (2017). Neural correlates of consumer buying motivations: a 7T functional magnetic resonance imaging (fMRI) study. Front. Neurosci. 11:512. 10.3389/fnins.2017.00512 28959182 PMC5603698

[B29] Gravetter F. J. Forzano L.-A. B. (2003). Research Methods for the Behavioral Sciences. Belmont, CS: Thomson.

[B30] Harris J. M. Ciorciari J. Gountas J. (2018). Consumer neuroscience for marketing researchers. J. Consum. Behav. 17, 239–252. 10.1002/cb.1710

[B31] Hoshi Y. Kobayashi N. Tamura M. (2001). Interpretation of near-infrared spectroscopy signals: a study with a newly developed perfused rat brain model. J. Appl. Physiol. 90, 1657–1662. 10.1152/jappl.2001.90.5.1657 11299252

[B32] Hubert M. Kenning P. (2008). A current overview of consumer neuroscience. J. Consum. Behav. 7, 272–292. 10.1002/cb.251

[B33] Kenning P. Plassmann H. (2005). NeuroEconomics: an overview from an economic perspective. Brain Res. Bull. 67, 343–354. 10.1016/j.brainresbull.2005.07.006 16216680

[B101] Kenning P. Plassmann H. Deppe M. Kugel H. Schwindt W. (2002). “Westfälische Wilhelms-Universität Münster,” in Neuroeconimic Research Reports: Neuromarketing (Münster, Germany), 1–26.

[B34] Kim J.-Y. Kim K.-I. Han C.-H. Lim J.-H. Im C.-H. (2016). Estimating consumers’ subjective preference using functional near infrared spectroscopy: a feasibility study. J. Near Infrared Spectrosc. 24, 433–441. 10.1255/jnirs.1242

[B35] Knutson B. Rick S. Wimmer G. E. Prelec D. Loewenstein G. (2007). Neural predictors of purchases. Neuron 53, 147–156. 10.1016/j.neuron.2006.11.010 17196537 PMC1876732

[B36] Kocsis L. Herman P. Eke A. (2006). The modified Beer-Lambert law revisited. Phys. Med. Biol. 51, N91–N98. 10.1088/0031-9155/51/5/N02 16481677

[B37] Koenigs M. Tranel D. (2007). Prefrontal cortex damage abolishes brand-cued changes in cola preference. Soc. Cogn. Affect. Neurosci. 3, 1–6. 10.1093/scan/nsm032 18392113 PMC2288573

[B38] Kohl M. Nolte C. Heekeren H. R. Horst S. Scholz U. Obrig H.. (1998). Determination of the wavelength dependence of the differential pathlength factor from near-infrared pulse signals. Phys. Med. Biol. 43, 1771–1782. 10.1088/0031-9155/43/6/028 9651039

[B39] Kopton I. M. Kenning P. (2014). Near-infrared spectroscopy (NIRS) as a new tool for neuroeconomic research. Front. Hum. Neurosci. 8:549. 10.3389/fnhum.2014.00549 25147517 PMC4124877

[B40] Kosslyn S. M. (1999). If neuroimaging is the answer, what is the question? Philos. Trans. R. Soc. Lond. B Biol. Sci. 354, 1283–1294. 10.1098/rstb.1999.0479 10466151 PMC1692630

[B41] Krampe C. Gier N. Kenning P. (2018a). “Beyond traditional neuroimaging: can mobile fNIRS add to NeuroIS?” in Information Systems and Neuroscience, (Springer), 151–157.

[B42] Krampe C. Strelow E. Haas A. Kenning P. (2018b). The application of mobile fNIRS to “shopper neuroscience”-first insights from a merchandising communication study. Eur. J. Mark. 52, 244–259. 10.1108/ejm-12-2016-0727

[B43] Kroger J. K. Sabb F. W. Fales C. L. Bookheimer S. Y. Cohen M. S. Holyoak K. J. (2002). Recruitment of anterior dorsolateral prefrontal cortex in human reasoning: a parametric study of relational complexity. Cereb. Cortex 12, 477–485. 10.1093/cercor/12.5.477 11950765

[B44] Kühn S. Strelow E. Gallinat J. (2016). Multiple “buy buttons” in the brain: forecasting chocolate sales at point-of-sale based on functional brain activation using fMRI. Neuroimage 136, 122–128. 10.1016/j.neuroimage.2016.05.021 27173762

[B45] Linzmajer M. Hubert M. Eberhardt T. Fojcik T. Kenning P. (2014). The effect of glucose consumption on customers’ price fairness perception. Schmalen. Business Rev. 2015, 7–49. 10.1007/bf03396917

[B46] Manes F. Sahakian B. Clark L. Rogers R. Antoun N. Aitken M.. (2002). Decision-making processes following damage to the prefrontal cortex. Brain 125, 624–639. 10.1093/brain/awf049 11872618

[B47] Masataka N. Perlovsky L. Hiraki K. (2015). Near-infrared spectroscopy (NIRS) in functional research of prefrontal cortex. Front. Hum. Neurosci. 9:274. 10.3389/fnhum.2015.00274 26029090 PMC4428134

[B48] McCormick P. W. Stewart M. Lewis G. Dujovny M. Ausman J. I. (1992). Intracerebral penetration of infrared light: technical note. J. Neurosurg. 76, 315–318. 10.3171/jns.1992.76.2.0315 1730963

[B49] Miyai I. Tanabe H. C. Sase I. Eda H. Oda I. Konishi I.. (2001). Cortical mapping of gait in humans: a near-infrared spectroscopic topography study. Neuroimage 14, 1186–1192. 10.1006/nimg.2001.0905 11697950

[B50] Naseer N. Hong K.-S. (2015). fNIRS-based brain-computer interfaces: a review. Front. Hum. Neurosci. 9:172. 10.3389/fnhum.2015.00172 25674060 PMC4309034

[B51] Plassmann H. O’Doherty J. Shiv B. Rangel A. (2008). Marketing actions can modulate neural representations of experienced pleasantness. Proc. Natl. Acad. Sci. U S A 105, 1050–1054. 10.1073/pnas.0706929105 18195362 PMC2242704

[B52] Plassmann H. Venkatraman V. Huettel S. Yoon C. (2015). Consumer neuroscience: applications, challenges, and possible solutions. J. Mark. Res. 52, 427–435. 10.1509/jmr.14.0048

[B53] Pochon J.-B. Levy R. Poline J.-B. Crozier S. Lehéricy S. Pillon B.. (2001). The role of dorsolateral prefrontal cortex in the preparation of forthcoming actions: an fMRI study. Cereb. Cortex 11, 260–266. 10.1093/cercor/11.3.260 11230097

[B54] Quaresima V. Ferrari M. (2016). Functional near-infrared spectroscopy (fNIRS) for assessing cerebral cortex function during human behavior in natural/social situations a concise review. Org. Res. Methods [Epub ahead of print]. 10.1177/1094428116658959

[B61] Rampl L. Eberhardt T. Schütte R. Kenning P. (2012). Consumer trust in food retailers: conceptual framework and empirical evidence. Int. J. Retail Distrib. Manag. 40, 254–272. 10.1108/09590551211211765

[B55] Raye C. L. Johnson M. K. Mitchell K. J. Reeder J. A. Greene E. J. (2002). Neuroimaging a single thought: dorsolateral PFC activity associated with refreshing just-activated information. Neuroimage 15, 447–453. 10.1006/nimg.2001.0983 11798278

[B56] Schaefer M. Rotte M. (2007). Favorite brands as cultural objects modulate reward circuit. Neuroreport 18, 141–145. 10.1097/wnr.0b013e328010ac84 17301679

[B57] Scholkmann F. Kleiser S. Metz A. J. Zimmermann R. Mata Pavia J. Wolf U.. (2014). A review on continuous wave functional near-infrared spectroscopy and imaging instrumentation and methodology. Neuroimage 85, 6–27. 10.1016/j.neuroimage.2013.05.004 23684868

[B58] Szalma J. L. Hancock P. A. (2011). Noise effects on human performance: a meta-analytic synthesis. Psychol. Bull. 137, 682–707. 10.1037/a0023987 21707130

[B59] Toga A. W. Thompson P. M. (2003). Mapping brain asymmetry. Nat. Rev. Neurosci. 4, 37–48. 10.1038/nrn1009 12511860

[B60] Torricelli A. Contini D. Pifferi A. Caffini M. Re R. Zucchelli L.. (2014). Time domain functional NIRS imaging for human brain mapping. Neuroimage 85, 28–50. 10.1016/j.neuroimage.2013.05.106 23747285

[B62] Werchan D. M. Collins A. G. Frank M. J. Amso D. (2016). Role of prefrontal cortex in learning and generalizing hierarchical rules in 8-month-old infants. J. Neurosci. 36, 10314–10322. 10.1523/JNEUROSCI.1351-16.2016 27707968 PMC5050327

[B63] Wilcox T. Biondi M. (2015). fNIRS in the developmental sciences. Wiley Interdiscip. Rev. Cogn. Sci. 6, 263–283. 10.1002/wcs.1343 26263229 PMC4979552

[B64] Worsley K. J. Friston K. J. (1995). Analysis of fMRI time-series revisited—again. Neuroimage 2, 173–181. 10.1006/nimg.1995.1023 9343600

[B65] Yoshino K. Oka N. Yamamoto K. Takahashi H. Kato T. (2013). Functional brain imaging using near-infrared spectroscopy during actual driving on an expressway. Front. Hum. Neurosci. 7:882. 10.3389/fnhum.2013.00882 24399949 PMC3871711

[B66] Zaltman G. (2000). Consumer researchers: take a hike! J. Consum. Res. 26, 423–428. 10.1086/209573

[B67] Zhao H. Tanikawa Y. Gao F. Onodera Y. Sassaroli A. Tanaka K.. (2002). Maps of optical differential pathlength factor of human adult forehead, somatosensory motor and occipital regions at multi-wavelengths in NIR. Phys. Med. Biol. 47, 2075–2093. 10.1088/0031-9155/47/12/306 12118602

