# Supplementary material for: The Application of Mobile fNIRS in Marketing Research—Detecting the “First-Choice-Brand” Effect
Source: Front Hum Neurosci. 2018 Nov 1;12:433. doi: 10.3389/fnhum.2018.00433 (PMC6222120; doi:10.3389/fnhum.2018.00433)
Supplement: Supplementary file 1 [file Data_Sheet_1.pdf]

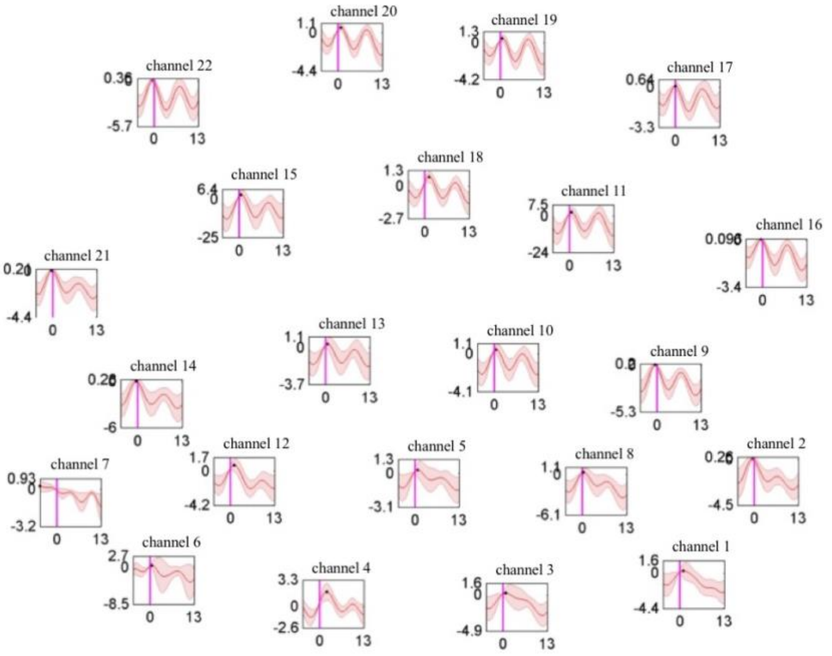

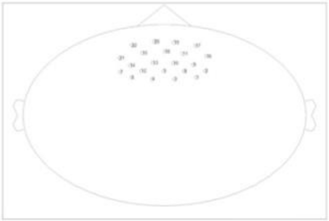

Appendix 1.1 – Representation of the oxygenated haemoglobin concentrations of target decisions (TD) and the standard deviation of every 22 channels respectively in the block integrating 80% TD-trails in the target brand group.

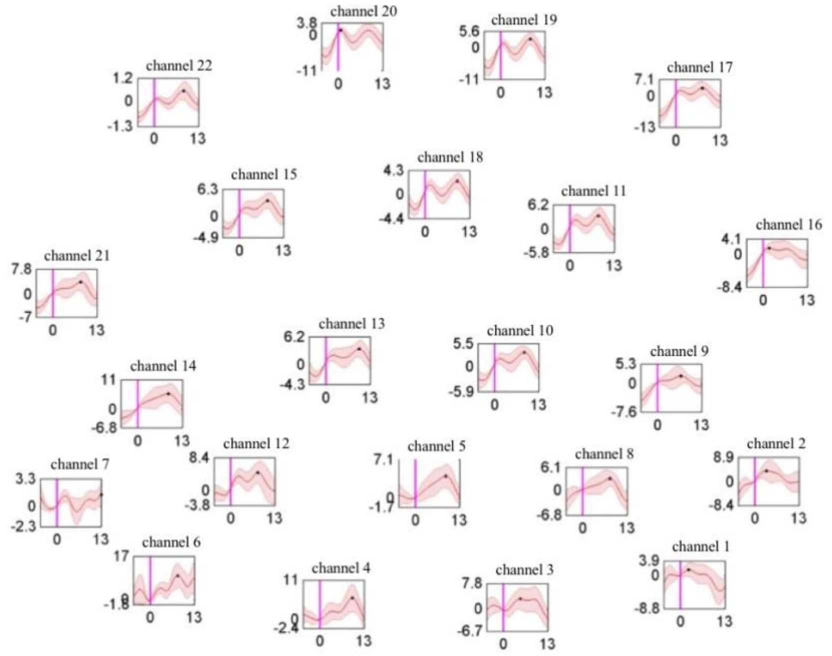

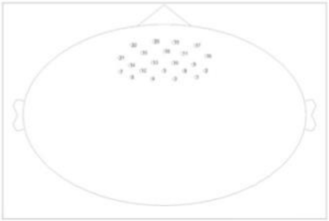

Appendix 1.2 – Representation of the oxygenated haemoglobin concentrations of target decisions (TD) and the standard deviation of every 22 channels respectively in the block integrating 20% TD-trails in the target brand group.

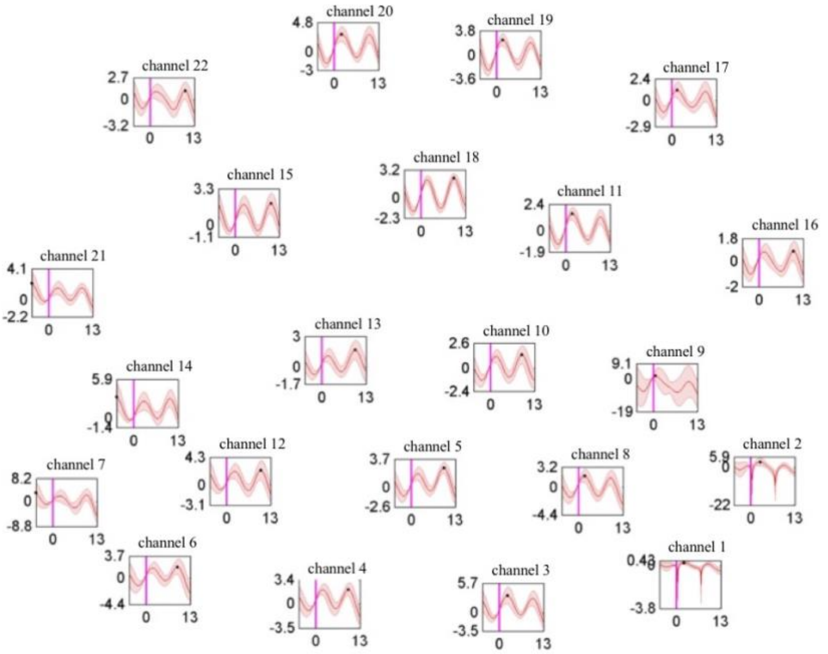

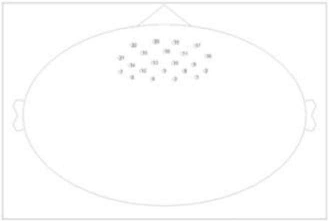

Appendix 1.3 – Representation of the oxygenated haemoglobin concentrations of target decisions (TD) and the standard deviation of every 22 channels respectively in the block integrating 80% TD-trails in the non-target brand group.

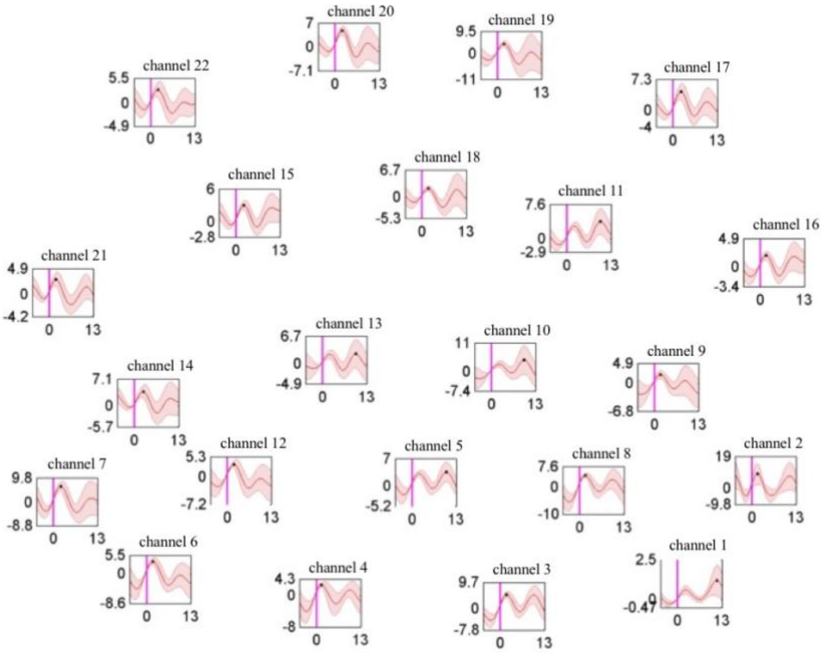

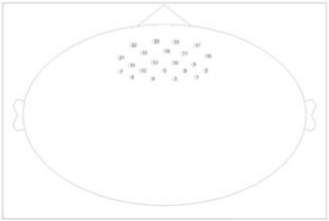

Appendix 1.4 – Representation of the oxygenated haemoglobin concentrations of target decisions (TD) and the standard deviation of every 22 channels respectively in the block integrating 20% TD-trails in the non-target brand group.
